# Innovative Reconstructive Management of Foot Macrodactyly in a Pediatric Patient: A Case Report

**DOI:** 10.7759/cureus.51398

**Published:** 2023-12-31

**Authors:** Ayyappan Thangavel, Abdulrahman Alsuwailim, Abdullah Albadran, Mazin Almousa, Saleh Al Molhim, Saleh K Alnafeesy, Abdulmohsen Almulhim

**Affiliations:** 1 Plastic Surgery, King Fahad Hospital, Al-Ahsa, SAU; 2 Urology, Almoosa Specialist Hospital, Al-Ahsa, SAU; 3 Medicine and Surgery, King Faisal University, Al-Ahsa, SAU; 4 Medicine and Surgery, Imam Abdulrahman Bin Faisal University, Dammam, SAU

**Keywords:** pediatric macrodactyly, foot reconstruction, pediatric orthopedic surgery, gigantism, plastic and reconstructive surgery, macrodactyly

## Abstract

Macrodactyly is a rare congenital anomaly characterized by disproportionate hypertrophy of one or more digits or the forefoot, involving some or all tissue types. It is nonhereditary and can present alone or alongside other deformities. Usually, macrodactyly is treated with amputation of the affected toe or finger to reduce the chance of recurrence. In this paper, we present the case of a child with macrodactyly who was treated successfully without amputation and instead with a reconstruction of the toe shape to resemble a near-natural-looking toe with intact functions.

The patient was a one-year-old female who presented with macrodactyly of her right great toe, right second toe, and forefoot. She had no history of other congenital deformities or systemic diseases. A reconstruction surgery was performed, which involved debulking the right great toe, right second toe, and forefoot. Also, it included the creation of the first web space and the restoration of the nailbed of the second toe. Postoperative follow-up revealed minimal complications. Thus, a second reconstructive surgery was performed, which included debulking and further reconstruction of the foot to improve the result.

Several techniques exist for the reduction of macrodactyly that can achieve optimal results. The choice of technique depends on the specifics of the case and the experience of the surgeon. We therefore hope our technique will be beneficial for the management of future cases of macrodactyly.

One year of follow-up after the second operation revealed maintained function and no regrowth recurrence.

## Introduction

Macrodactyly is a rare congenital anomaly characterized by disproportionate hypertrophy of one or more digits as well as the forefoot. It can involve all tissue types (bone, nerve, vessels, fat, skin, etc.). It is nonhereditary and can present alone or alongside other deformities [1]. The prevalence of macrodactyly is one per 100,000-125,000 childbirths, and it occurs more often in hands than feet [2, 3]. Usually, macrodactyly is unilateral and affects more than one digit, with the second or third digit typically involved [3]. Although macrodactyly affects all tissues in the digit, growth often varies between tissue types. Some instances exhibit predominantly soft tissue overgrowth, whereas others exhibit a greater increase in bone growth than soft tissue growth [1, 2]. The etiology of macrodactyly is believed to be associated with several gene mutations, including the mosaic PIK3CA mutation, AKT1, and PTEN mutation [4]. Normally, mirror-image symmetry in limbs is fine in the vertebrate phenotype. Genetic and epigenetic factors regulate the differentiation, patterning, and development of the embryo and fetus [5]. Diagnosing a case of local gigantism should be reserved for patients with isolated congenital overgrowth affecting all tissue types, but the clinical presentation and natural history of macrodactyly can vary greatly among patients [2].

Surgery is the mainstay of treatment for macrodactyly. Surgical options vary considerably and can be chosen based on the extent, type, rate of overgrowth, family expectations, as well as the surgeon's experience and preference. The intention of undergoing surgery is to improve the function and cosmetic appearance of the affected limb. Treatment options include epiphysiodesis, which halts growth in the affected area; osteotomies, which reshape bones; debulking, reducing excess tissue; toe transfers for reconstruction; and amputations as a last resort. Amputation is widely performed for macrodactyly. However, this technique has been highly reported for the risk of recurrence, skin necrosis, chronic pain, and postoperative infections, hindering both function and cosmetic appearance. Our reconstructive approach enhances functional and cosmetic outcomes by utilizing skin flaps and local parts, presenting a more nuanced and patient-centric approach to managing macrodactyly [6, 7]. This study’s purpose is to share our experience in diagnosing and treating a case of macrodactyly in a one-year-old female child with two years of follow-up.

## Case presentation

**Figure 1:**
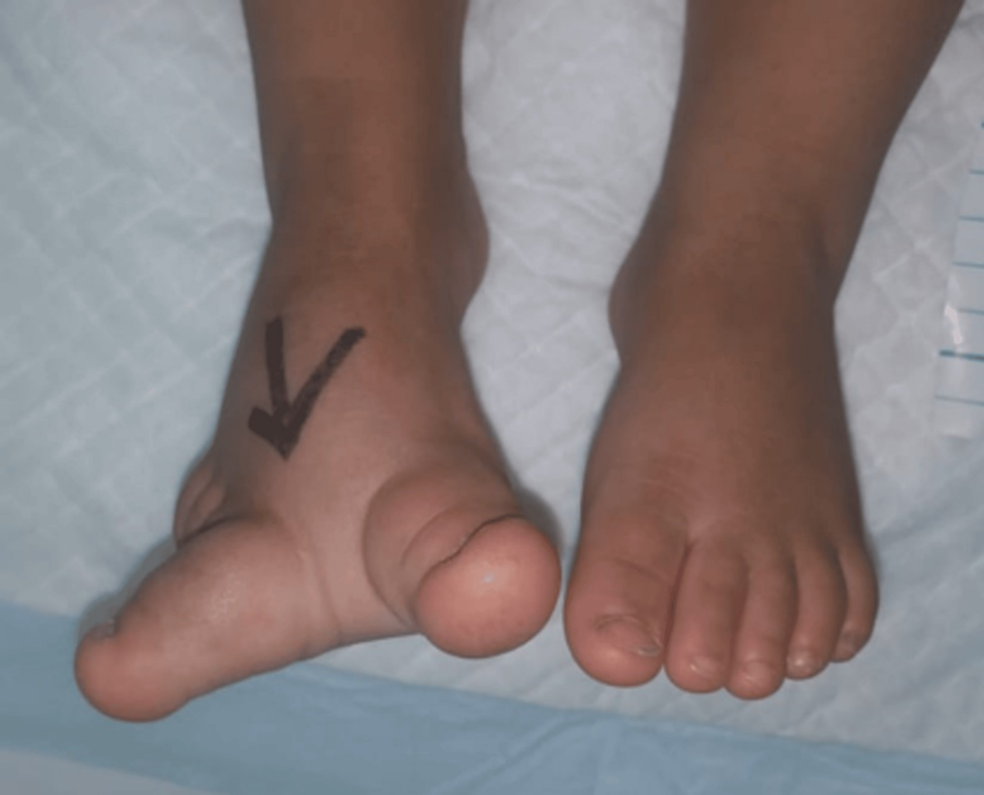
The patient's right foot before the operation

A one-year-old female patient presented with congenital enlargement of her first toe, second toe, and right forefoot. (Figure 1).

She exhibited no evidence of other congenital deformities or systemic diseases. Genetic testing for the cause of macrodactyly was not conducted on our patient. The patient was born at full-term gestation with normal gestation and delivery.

**Figure 2:**
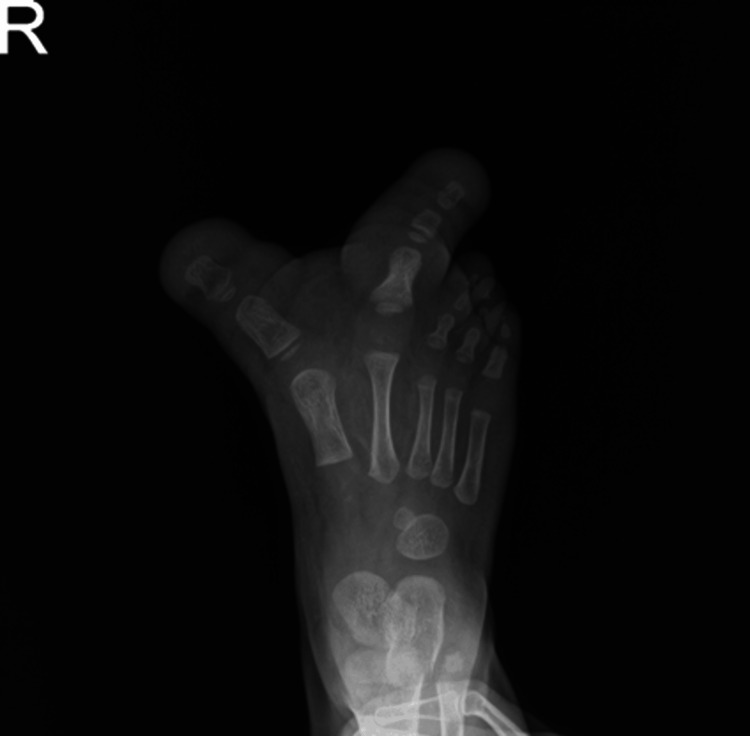
X-ray of the right foot before the operation

Physical examination revealed an enlarged right forefoot and the right first and second toes were larger than normal and curved outward. There were no associated congenital deformities. Her vital parameters were normal, and the lab results were appropriate for surgery. An X-ray showed enlargement of the proximal and distal phalanges (Figure 2).

**Figure 3:**
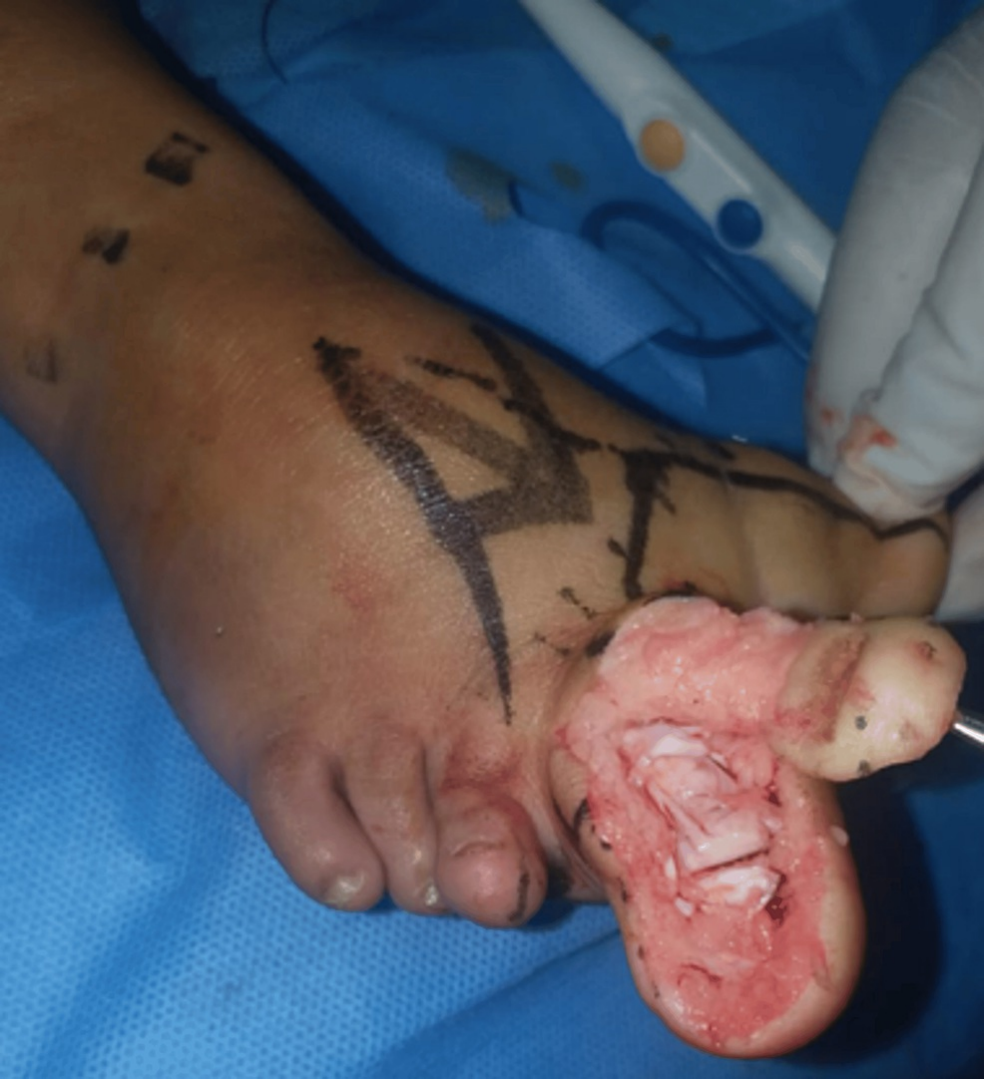
The patient's right foot during the operation

Surgery was conducted for a second toe reconstruction and debulking of the great toe and forefoot under general anesthesia in a well-sterilized environment. The forefoot was reconstructed by debulking both the dorsal and plantar aspects and debulking the great toe on its medial aspect (Figure 3).

**Figure 4:**
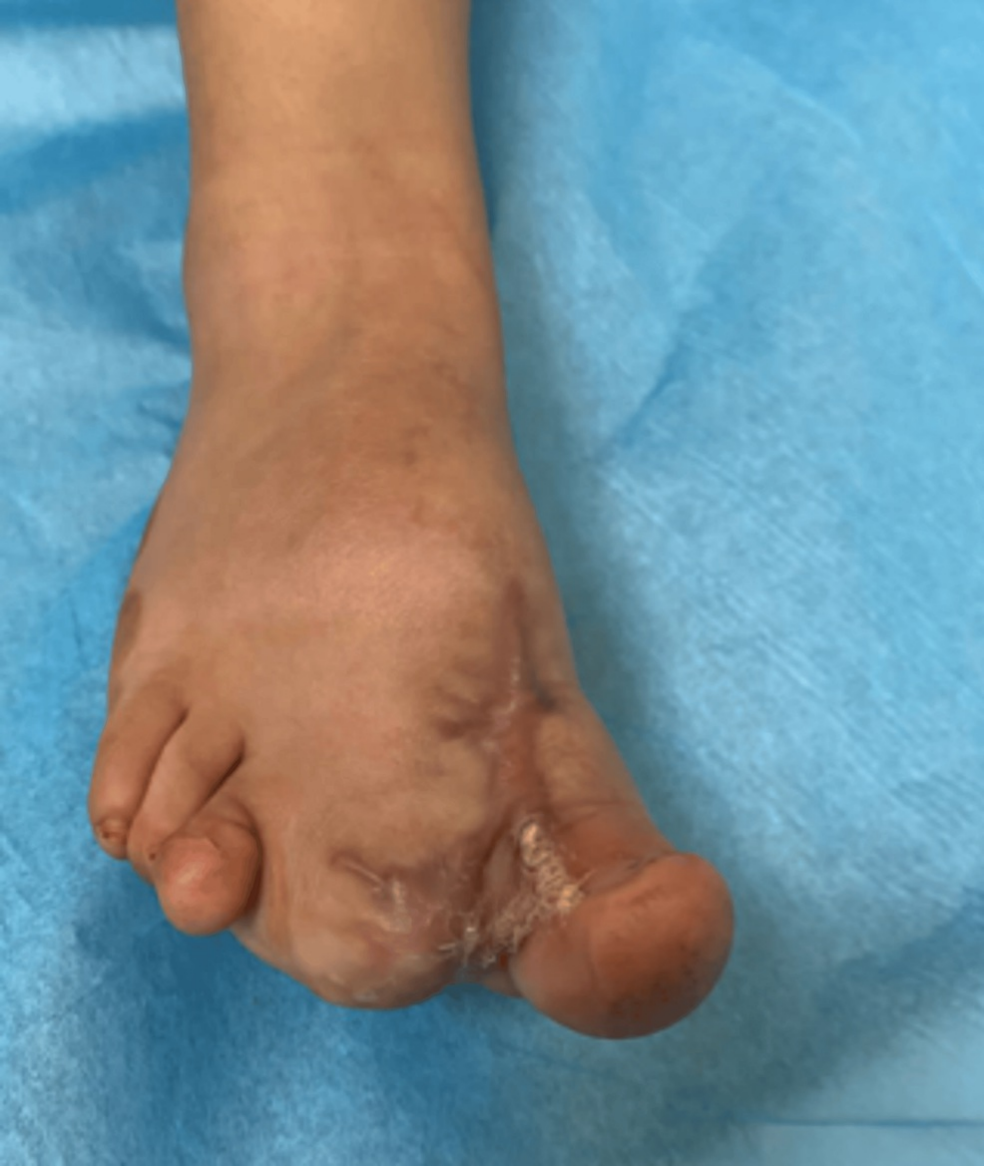
The patient's foot during the follow-up six months after the first operation

The residual raw areas were covered by local advancement flaps from the dorsal aspect of the medial right foot. The reconstruction of the second toe was more complex and involved the creation of the first web space with an island skin flap based on a de-epithelized dermal pedicle. The nail bed was then transported proximally based on the de-epithelized dermal pedicle. The distal phalanx was removed, the proximal phalanx was resized to simulate the length of a normal toe, and the growth center was cauterized. The nailbed was repositioned on the refashioned proximal toe, unfolding the dermal pedicle in layers like an accordion. Sutures were then applied to approximate the great toe and the second toe. This reduced the cleft foot deformity to mimic normal toe alignment. A slab was applied below the knee after the dressing. The wounds were sutured with a 5.0 nylon suture. Postoperatively, the patient had no infection or ischemia, and all the reconstructed parts survived well (Figure 4).

The patient stayed in the hospital for a few hours after the operation, then was discharged home on paracetamol and Augmentin.

**Figure 5:**
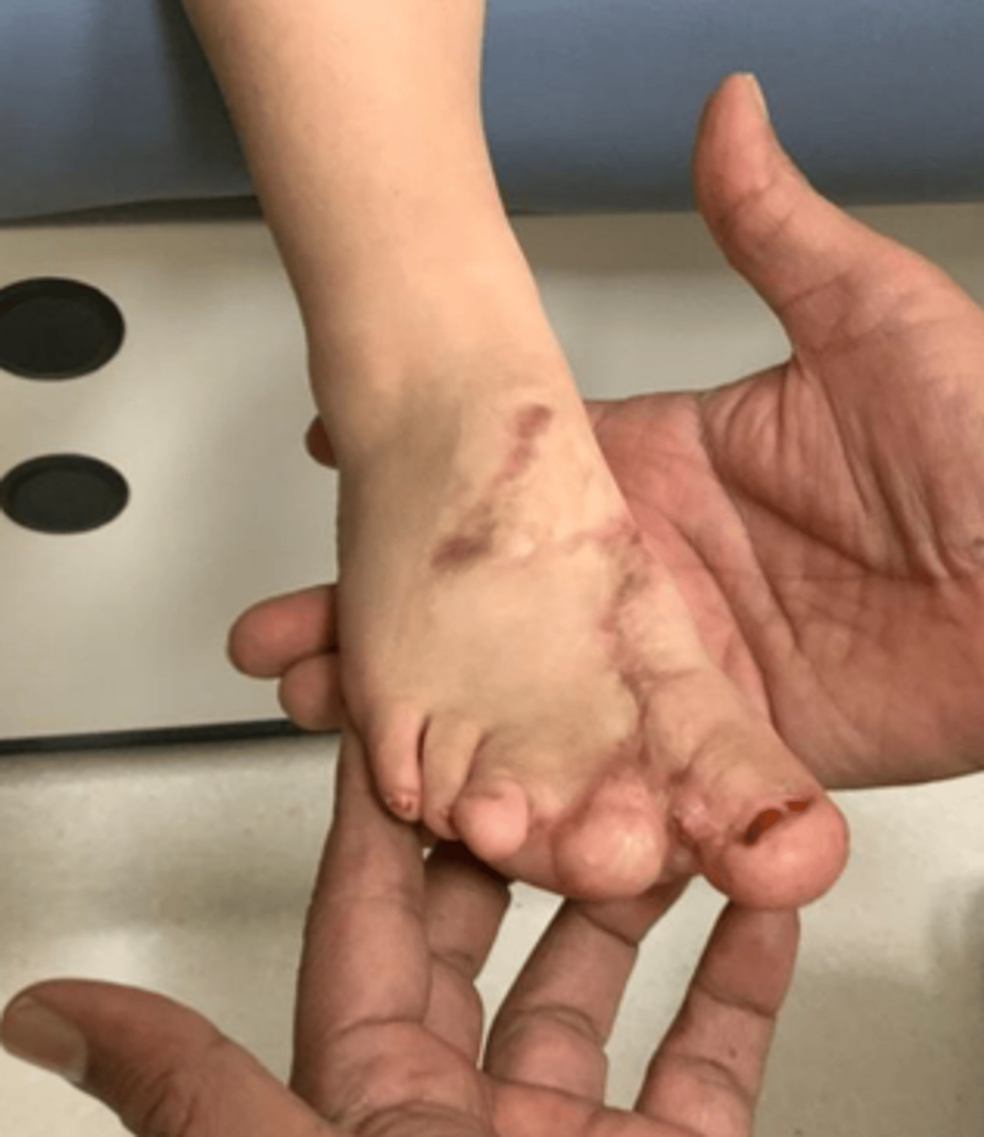
The patient's foot during the follow-up six months after the second operation

The follow-up after six months revealed a hypertrophic scar and the need for further debulking. Also, we noticed that the attempt at nail bed repositioning and reconstruction was unsuccessful. We therefore arranged for a second surgery. The surgery started by developing multiple local skin flaps from the sole aspect of the great toe, distal foot, and midfoot. Subsequently, the surgeon performed a radical debulking of the ventral aspect of the great toe distal to the proximal foot through the midfoot. Complete hemostasis was obtained, after which skin cover was obtained by re-draping predesigned skin flaps. Corrugated drains were placed between interrupted simple skin sutures using a 3.0-cuting nylon suture. A dressing was applied in layers. The first layer was sofra-tulle, and the second was gauze pieces. Gauze pieces were also put in the interdigital spaces to prevent maceration before a fluffy cotton roll and a snug bandage were applied. Finally, the foot was encased in a plaster of Paris slab, which was applied to keep the ankle at 90° for six weeks. Six months after the second operation, a follow-up revealed a stable toe that maintained normal functionality and still looked nearly normal (Figure 5).

## Discussion

Macrodactyly is a rare congenital deformity that affects one or more digits of the hand or foot, involving some or all tissue types. Many options exist for treating macrodactyly. The optimal treatment result is to have a functioning toe, a normal gait, the ability to wear shoes, and an appearance that is as natural as possible [2]. Surgical interventions seek to enhance functionality and reduce the deformity and symptoms that accompany the condition. Various surgical methods are implemented, such as fat mass reduction by debulking and dissecting soft tissue, osteotomy or ostectomy, epiphysiodesis, and amputation. No standard surgical technique exists for treating macrodactyly yet. The choice conventionally combines an assortment of surgical techniques appropriate to the specifics of each case. The surgeons’ expertise, skills, and availability of the hospital’s equipment must be taken into consideration for the successful treatment of macrodactyly. Treating macrodactyly is, therefore, a considerable challenge for surgeons [5].

Deciding an appropriate time for a surgical intervention depends on the severity of the macrodactyly and the patient’s age. Some researchers recommend surgery before the child begins to walk [8, 9]. However, others suggest postponing intervention until the child is three to four years old or even after adolescence [10]. Surgeons should consider three crucial elements before deciding whether to perform or postpone the surgery: the physical element, the physiological element, and the expected outcome. During their cognitive development, a child with macrodactyly will often struggle to understand why they endure such an impairment, which is very difficult for the parents to explain when the child inevitably asks about it [11]. Therefore, considering the functional and psychological aspects, the surgery was performed when the age of the patient in this case was one.

Ray amputation is a widely performed surgical technique for progressive macrodactyly [12, 13]. Yet this technique has been highly reported for the risk of recurrence, skin necrosis, chronic pain, and infection after surgery [14]. Another common technique is ray reduction. The strong disadvantage of these two techniques is the aesthetic outcome. They involve removing one or more segments of the patient’s foot. Losing a digit or even a toenail would hinder the patient’s ability to do things like wear open-toed shoes or apply nail polish. It may also negatively impact the patient’s feelings or limit their socialization or participation in some activities, such as swimming. Furthermore, Hardwicke et al. (2013) reported a severe psychological impact that accompanies macrodactyly, particularly for school-age children [3]. Josh et al. (2021) reported a similar case to ours, where the patient presented with macrodactyly in the foot digits. They performed reconstructive surgery by removing the medial phalanx of the digit, debulking soft tissue, and shortening the tendons. They achieved successful and satisfactory results [15].

In our case, we performed reconstructive surgery on the foot. We maintained the toe functions and made the shape of the feet and nails nearly normal. We did this without amputating any of the digits, only performing debulking and an ostectomy by removing parts of the soft tissue, bone, and cartilage, thereby, reducing the size of the foot to fit into a shoe. We have planned to follow the patient every year as she might require additional debulking procedures in the growth phase.

## Conclusions

In this case, reconstructive surgery for a one-year-old female with macrodactyly of the right foot was done in an attempt to restore normal foot contour and function without the need to amputate any of the digits. We hope that this type of surgery will help reduce the psychological and functional impact that accompanies this kind of disorder.
